# Hypoglycemic Effects in Alloxan-Induced Diabetic Rats of the Phenolic Extract from Mongolian Oak Cups Enriched in Ellagic Acid, Kaempferol and Their Derivatives

**DOI:** 10.3390/molecules23051046

**Published:** 2018-04-30

**Authors:** Peipei Yin, Yu Wang, Lingguang Yang, Jinling Sui, Yujun Liu

**Affiliations:** 1 College of Biological Sciences and Biotechnology, Beijing Forestry University, Beijing 100083, China; happy62889@126.com (P.Y.); yanglingguangxdjqz@163.com (L.Y.); 2 School of Nature Conservation, Beijing Forestry University, Beijing 100083, China; m15366119695@163.com

**Keywords:** Mongolian oak cups, ethanol crude extract (ECE), ellagic acid- and kaempferol-derivatives, alloxan-induced rats, type 1 diabetes, hypoglycemic effect

## Abstract

Our previous reports showed that crude extract prepared with 50% ethanol (ethanol crude extract, ECE) from Mongolian oak cups possessed excellent in vitro antioxidant capacities as well as inhibitory activities against α-glucosidase, α-amylase and protein glycation caused by its enrichment in phenolics, including mainly ellagic acid, kaempferol and their derivatives. Nevertheless, few in vivo studies on antidiabetic activities of these phenolics were conducted. The present study investigated hypoglycemic effects with normal and diabetic rats being administrated orally without or with ECE at 200 and 800 mg/kg for 15 days. In normal rats, no significant differences were exhibited after ECE administration in body weight, fasting blood glucose level, levels of cholesterol, triglyceride, LDL and AST in serum, organ indexes, and levels of GSH and MDA in organs. In diabetic rats, the fasting blood glucose level, indexes of heart and liver, and levels of cholesterol and triglyceride in serum and MDA in heart tissue were significantly decreased. Moreover, HDL levels in serum and SOD activities in the four organs of diabetic rats were significantly improved after ECE administration at 800 mg/kg. Thus, in addition to inhibiting α-glucosidase, α-amylase and protein glycation reported previously, oak cups might contain novel dietary phytonutrients in preventing abnormal changes in blood glucose and lipid profile and attenuating oxidant stress in vivo. The results also implied that it is ellagic acid, kaempferol and their derivatives enriched in ECE that might play vital roles in managing type 1 as well as type 2 diabetes.

## 1. Introduction

Diabetes, caused by inherited and/or acquired deficiency in production of insulin by the pancreas (type 1) or by ineffectiveness of insulin (type 2), is one of the most burdensome and costly chronic disease in the world [1,2,3]. According to the International Diabetes Federation, 382 million people are suffering from diabetes and diabetes patients will increase to about 592 million by 2035 [4]. Diabetes can be caused by a number of biological factors, of which insulin resistance and deficiency are both related to hyperglycemia and hyperlipidemia [5,6].

In addition to death, diabetes also lead to many chronic conditions like neuropathy, nephropathy, and various vascular diseases associated with heart, kidney, brain, peripheral blood vessels and retinopathy [3,7]. It has been reported that oxidative stress leads to excessive production of free radicals which are implicated in pathogeneses of diabetes and its complications [7,8]. Moreover, the excessive generation of free radicals associated with diabetes will result in oxidative damage, particularly in kidney, liver, eyes, small and large blood vessels, and immunological and gastrointestinal systems [2,3]. Although insulin and other artificially synthesized anti-diabetes drugs like acarbose and voglibose are effective for the management of diabetes, they are far from satisfying the urgency for their enormous costs or undesirable side effects [1,9]. Thus, searching alternative anti-diabetic natural products from plants has received great attention.

Mongolian oak, belonging to the Fagaceae, is largely distributed in China, Russia, Mongolia, Japan and Korea, and its acorns were popularly used for foods. In China, they are mainly found in Heilongjiang, Jilin and Liaoning [1]. It has been reported that extracts of leaves, acorns or woods from oak trees possessed numerous kinds of biological activities, including antioxidant, anti-inflammatory, anticarcinogenic, antidiabetic and antimicrobial activities [10,11,12]. Moreover, our two previous reports showed that phenolic extracts of oak cups were rich in phenolics (mainly ellagic acid, kaempferol and their derivatives), with high antioxidant and antidiabetic activities in vitro [1,13]. They were found to be expressly effective in controlling diabetes and its complications by inhibiting activities of α-glucosidase and α-amylase and formation of advanced glycation end-products. However, to the best of our knowledge, there was no in vivo report on antidiabetic activity of phenolic extracts from oak cups. Therefore, the potentially anti-diabetic effects of phenolic extracts from oak cups required further in vivo validation.

As a strong oxidant, alloxan is widely used in experimental animals to induce insulin-dependent diabetes (type 1). It works by increasing generation of reactive oxygen species from metabolic reactions in the body, together with massive increase of cytosolic calcium concentration, and it can rapidly cause destruction of pancreatic β-cells [14,15,16]. The objective of this study was to evaluate hypoglycemic effects of phenolic extracts prepared with the optimal 50% ethanol from Mongolian oak cups [12] with normal and alloxan-induced diabetic rats.

## 2. Results and Discussion

### 2.1. Effects of ECE on Fasting Blood Glucose of Normal and Diabetic Rats

**Figure 1:**
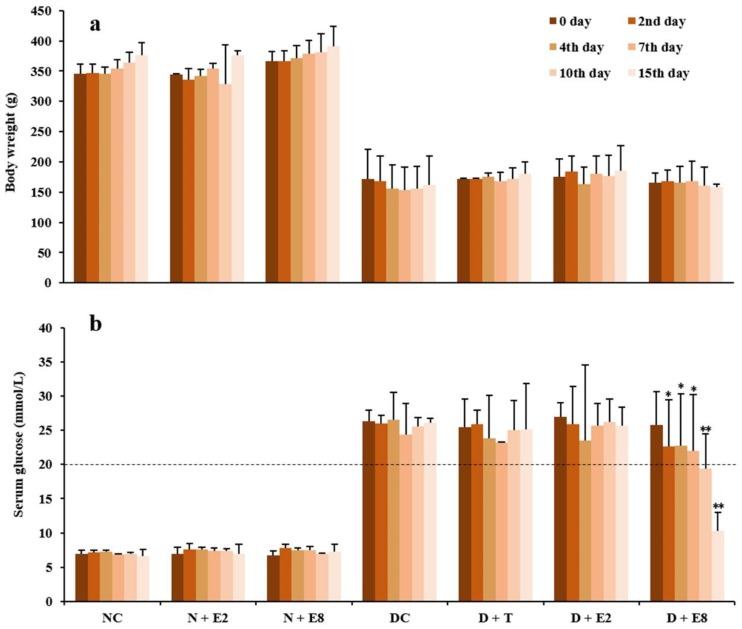
Effects of ECE on body weights (**a**) and fasting blood glucose levels (**b**) in normal and alloxan-induced diabetic rats. Results were presented as mean ± SD with five independent experiments (*n* = 5). Normal rats treated with ECE (N + E2 and N + E8) were compared with those of the normal control (NC), whereas alloxan-induced diabetic rats further treated with tolbutamide (D + T) or ECE (D + E2 and D + E8) were compared with those of the diabetes control (DC) on the same day. Note that both significant (* *p* < 0.05) and extremely significant (** *p* < 0.01) differences were existed only in D + E8 in comparison with DC on the same day. N, normal; C, control; D, diabetes; T, tolbutamide at 100 mg/kg; E2, ECE at 200 mg/kg; E8, ECE at 800 mg/kg.

Diabetes is characterized by hyperglycemia with an increase of glucose in blood. Alloxan is a classical diabetogenic chemical which exerts selective cytotoxic influences on pancreatic β-cells, resulting in destruction of β-cells and type 1 diabetes. Figure 1 shows changes in body weights and fasting blood glucose levels of individual groups of rats within 15 days after a model of diabetic rats being established. It is well known that loss of body weight is one of the most intuitive indicators of diabetes [5], which was clearly evidenced by our data shown in Figure 1a that body weights of all the diabetic groups of rats (i.e., DC, D + T, D + E2 and D + E8) were only roughly half of those of the normal groups (i.e., NC, N + E2 and N + E8), with no significant differences within both the three normal and four diabetic groups. Meanwhile, in normal rats, there were no significant differences in levels of fasting blood glucose (Figure 1b) between the normal control (NC) and ECE treatments both at 200 (N + E2) and 800 (N + E8) mg/kg, suggesting that ECE gave rise to no influence on not only body weight but also fasting blood glucose of normal rats.

Levels of fasting blood glucose of diabetic rats which were injected with alloxan at a dose of 150 mg/kg one week before the zero day (i.e., 0 day) were all higher than 20 mmol/L as indicated by the dashed line (Figure 1b; DC). These high levels of fasting blood glucose, together with loss of weights (Figure 1a; DC), low survival rates, polyphagia, polydipsia and polyuria (data not shown), could confirm that a model of diabetic rats had been successfully established [6]. Moreover, by comparing data of D + T rats with those of DC rats in Figure 1b, it is obvious that there was no significant decrease of fasting blood glucose after oral administration of tolbutamide at a dose of 100 mg/kg for as long as 15 days. Ratzmann et al. [17] reported that tolbutamide was effective in management of type 2 diabetes, since the pancreas must synthesize insulin for tolbutamide functioning. In our experiment, tolbutamide exhibited no effect on fasting blood glucose in diabetic rats (Figure 1b; D + T), implying that the pancreatic β-cells might be damaged too severely by alloxan to synthesize insulin. Therefore, the model established was more likely to be type 1 diabetes [14].

In diabetic rats, oral administration of ECE at 200 mg/kg (Figure 1b; D + E2) even for 15 days could not reduce fasting blood glucose and there were no significant differences compared with those of the DC rats at each of the same days, which probably due to the still short time of ECE treatment as well as the relatively lower dosage. Nevertheless, oral administration of ECE at 800 mg/kg (Figure 1b; D + E8) caused significant declines of the fasting blood glucose compared to those of the DC rats at the correspondingly same day from the 2nd to the 7th day (*p* < 0.05) and extremely significant decreases since the 10th day (*p* < 0.01). The results demonstrated that ECE could effectively reduce the fasting blood glucose of diabetic rats, with no side-effects on both body weights (Figure 1a; N + E2 and N + E8) and fasting blood glucose (Figure 1b; N + E2 and N + E8) in the normal groups of rats.

The results shown in Figure 1 were consistent with one of our previous reports that phenolic extracts from burs of chestnut that belongs to the family Fagaceae also exhibited a hypoglycemic effect in diabetic rats [6]. In addition, Hou et al. [18] and Arumugam et al. [19] also reported that extracts from several plant species in the family Fagaceae were effective in reducing blood glucose of diabetic rats. Recently, Encarnação et al. [20] and Mollica et al. [21,22,23] reported that *Anacardium occidentale* stem bark, *Capparis spinosa* flower buds and *Juglans regia* leaf are rich in phenolics with high antioxidant activity and at the same time exhibited strong hypoglycemic activity. On the other hand, it is commonly known that alloxan potentially damages the pancreatic β-cells because it led to antioxidant competence of the β-cells being much lower than cells in other tissues did [24]. Therefore, as the ECE, an extract prepared with 50% ethanol from Mongolian oak cups, is abundant in phenolics with superior antioxidant activity [1,12], its hypoglycemic activity might due to its antioxidant activity to enhance pancreatic β-cells viability of alloxan-induced diabetic rats in this study. Furthermore, a decline of rapidly increased postprandial hyperglycemia through inhibition of enzymes is used in the management of type 2 diabetes, with the main enzymes being α-glucosidase and α-amylase [25,26,27]. Our previous study showed that ECE exhibited drastic inhibitory activities against α-glucosidase and α-amylase [12], indicating that ECE could also contribute to treatment of type 2 diabetes. All the aforementioned results demonstrated for the first time that oak cups, being underutilized byproducts, held huge potential to be developed into a novel dietary phytonutrient for management of both types 1 and 2 diabetes.

Moreover, one of our previous report [1] showed that 24 phenolics were identified from the ECE by UPLC-MS/MS, and ellagic acid- and kaempferol-derivatives were the main phenolics. Recently, antidiabetic effects of ellagic acid, kaempferol and their derivatives had also been reported in diabetic model [28,29,30,31,32]. Ellagic acid and its derivatives could prevent diabetes through inhibition of insulitis [31], and kaempferol possesses the beneficial effects of preserving pancreatic β-cell mass and function [28]. Thus, we speculated that it is ellagic acid, kaempferol and their derivatives enriched in ECE that might play vital roles in managing type 1 as well as type 2 diabetes.

### 2.2. Lipid Profiles and AST Levels in Serum of Normal and Diabetic Rats

#### 2.2.1. Effects of ECE on Serum Lipid Profiles

A number of studies reported that streptozocin- or alloxan-induced diabetes results in dyslipidemia, including increases in cholesterol, triglyceride and LDL and decrease in HDL [33,34], which would enhance the risk of acquiring cardiovascular disease [35]. As shown in Table 1, compared to those in the NC rats, cholesterol and triglyceride increased extremely significantly, and HDL decreased and LDL increased both significantly in DC rats, being in agreement with previous studies [33,34].

In diabetic rats, ECE treatment for 15 consecutive days could decrease cholesterol, triglyceride and LDL levels, and increase HDL level in a dose-dependent manner compared to those of the DC rats. It must be emphasized that compared to those in NC rats there existed no significant difference in cholesterol, triglyceride and HDL but a significant increase only in LDL in D + E8 rats, and compared to DC rats there existed significant differences in cholesterol, triglyceride and HDL but no significant difference in LDL in D + E8 rats and significant difference in D + E2 rats only in triglyceride. These results suggest that ECE could improve the lipid profile of alloxan-induced diabetic rats, which may lead to a decrease of the risk of diabetic complications [7]. It is also worth noting that no significant differences was observed in cholesterol and triglyceride, while there existed significant decreases in HDL and extremely significant increases in LDL in both N + E2 and N + E8 rats compared to those in the NC rats, which would cause negative influences upon the treated rats thus need to be investigated further.

#### 2.2.2. Effects of ECE on Serum AST Levels

**Figure 2:**
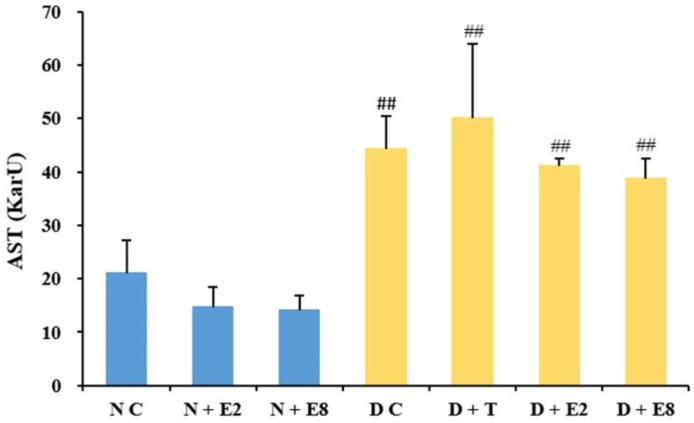
Effects of ECE on AST levels in plasma of normal (blue bars) and diabetic (yellow bars) rats. Results were presented as mean ± SD with five independent experiments (*n* = 5). ^##^ indicates extremely significant (*p* < 0.01) between the normal controls (NC) and all other groups, respectively. Note that no significant difference was observed among the three normal and the four diabetic groups of rats. N, normal; C, control; D, diabetes; T, tolbutamide at 100 mg/kg; E2, ECE at 200 mg/kg; E8, ECE at 800 mg/kg.

AST is one of the biochemical indicators of liver function [36]. Figure 2 exhibited AST levels in both normal and diabetic rats without or with ECE treatments. The AST level extremely significantly increased after the alloxan induction compared to that in NC rats, and showed dose-dependent decreases both in N + E2 and N+E8 rats compared to NC rats and in D + E2 and D + E8 rats compared to DC rats, although no significant differences were observed within the three normal and the four diabetic groups of rats. The results demonstrated that ECE exerted no significant influences on AST level in both normal and diabetic rats, while ECE administration did decrease, although with no significant differences, AST levels of both normal and diabetic rats to some extent during the course of experiment, which likely revealed that ECE might further improve liver conditions in normal rats and alleviate liver damages caused in diabetic rats. Similarly, the probiotic *Lactobacillus paraplantarum* BGCG11 has also showed the ability to attenuate liver damage by reducing AST level in diabetic rats [37]. Therefore, as an oak industry byproduct, oak cups might hold certain potential to be developed into therapeutic agents for treatment of liver disease associated with diabetes.

### 2.3. Effects of ECE on Four Organ Indexes of Normal and Diabetic Rats

**Figure 3:**
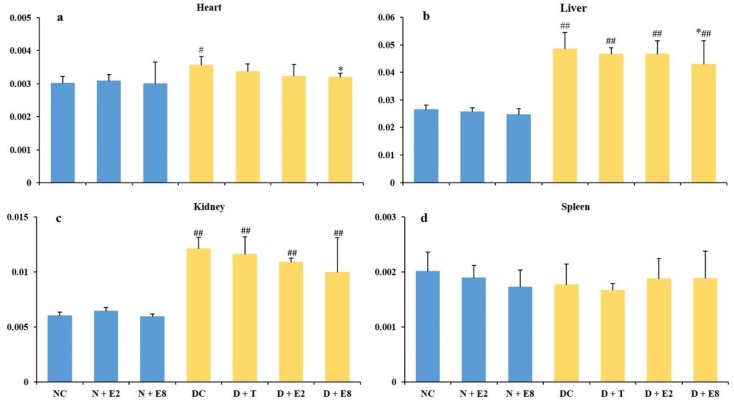
Effects of ECE on organ indexes (**a**, heart; **b**, liver; **c**, kidney; **d**, spleen) of normal (blue bars) and diabetic (yellow bars) rats. Results were presented as mean ± SD with five independent experiments (*n* = 5). ^#^ and ^##^ indicate significant (*p* < 0.05) and extremely significant (*p* < 0.01) differences between the normal controls (NC) and all other groups, and * indicates significant (*p* < 0.05) differences between the diabetic controls (DC) and all other diabetic rats (D + T, D + E2 and D + E8), respectively. N, normal; C, control; D, diabetes; T, tolbutamide at 100 mg/kg; E2, ECE at 200 mg/kg; E8, ECE at 800 mg/kg.

Organ indexes could reveal damage degrees of tissues in diabetic rats. As shown in Figure 3, in normal rats, ECE exhibited no significant influences on all the four organs (Figure 3a–d; N + E2 and N + E8 vs. NC), indicating that ECE caused no tissue damages in normal rats. In contrast, the organ indexes of heart, liver and kidney in DC rats all increased and were either significantly (heart) or extremely significantly (liver and kidney) different from those of the NC rats (Figure 3a–c; DC vs. NC), while there existed no significant difference between the two groups of rats with respect to the spleen index (Figure 3d; DC vs. NC), indicating that alloxan induction might lead to damages on heart, liver and kidney, but brought about no significant influence on spleen.

After ECE administration, indexes of heart, liver and kidney in diabetic groups all decreased to different extents in a dose-dependent manner (Figure 3a–c; D + E2 and D + E8 vs. DC), and only the indexes of heart (Figure 3a) and liver (Figure 3b) in D + E8 rats exhibited significant difference from those of the DC rats, but no significant differences were found in the indexes of kidney and spleen between D + E8 and DC groups, indicating that ECE gave rise to greater influence upon heart and liver than kidney and spleen. Furthermore extremely significant differences were observed between all the ECE-treated diabetic rats (D + E2 and D + E8) and the NC rats in liver and kidney (Figure 3b,c) but not in heart and spleen (Figure 3a,d). Considering the significant and the extremely significant differences existed between DC and NC rats in the heart and liver indexes in respective (Figure 3a,b; D + E8 vs. DC vs. NC), ECE might even relieve the damage to certain extent to these two organs caused by the alloxan induction in addition to its contribution to management of both types 1 and 2 diabetes as analyzed in the above subsection.

It had been reported that hyperglycemia could increase oxidative stress and lipid peroxidation and further resulted in tissue damage [7,38]. Therefore, it is possible to deduce from these results that the oxidative stress and lipid peroxidation increased thus caused damage in tissues of diabetic rats after alloxan-induction and ECE could reduce the damage of organs, especially in heart and liver, probably via its higher antioxidant activities [13].

### 2.4. Antioxidant Activities of ECE in Organs of Normal and Diabetic Rats

#### 2.4.1. Effects of ECE on GSH Levels

GSH is mainly synthesized in liver and is an important non-enzymatic antioxidant and free radical scavenger of cells [8]. It is one of the indicators of the in vivo antioxidant defense system, and a sharp reduction of GSH levels can usually be observed in diabetic rats [39]. However, as shown in Table 2 (DC vs. NC), increases of GSH levels were observed in all the four organs after alloxan stimulation, but only with those in heart and spleen being significant. The reason for these increases might be the experiment period being too short [40], that is, 7 days might be not long enough for the decrease of GSH levels to occur in tissues of diabetic rats, especially in heart and spleen. For more details of the data in Table 2, it is obvious that no significant difference was found in all the four organs among NC, N + E2 and N + E8 rats, indicating that ECE exerted no significant influence on GSH levels in normal rats. In liver, significant reduction from both of NC and DC was observed only in D + E8 rats. Furthermore, ECE administration to the diabetic rats led to reductions of GSH levels in three organs, with kidney as an exception at the lower dose of 200 mg/kg (i.e., D + E2; 1.43 ± 0.36 μmol/g prot) which was little higher than that of DC (1.38 ± 0.33 μmol/g prot). Moreover, reductions in hearts of both D + E2 and D + E8 rats were extremely significant from that of DC rats, and those in heart and liver of D + E8 rats were even significant from that of NC rats. These results suggested that the reduceed GSH in diabetic rats could be the result of decreased synthesis and/or increased degradation of GSH by oxidative stress in diabetes [41]. Similar results were found in *Cydonia oblonga* (Rosaceae) leaves, *Helianthus tuberosus* (Asteraceae) tubers, and *Allium porrum* (Liliaceae) bulbs as well, which were also used as a folk remedy for the treatment of diabetes in Turkey [40].

#### 2.4.2. Effects of ECE on MDA Levels

In diabetes, chronic hyperglycemia can induce carbonyl stress, which would lead to the increase of lipid peroxidation [42]. MDA is one of the most prevalent byproducts of lipid peroxidation during oxidative stress, therefore the content of MDA could be used as an index of the lipid peroxidation degree [15,43]. As listed in Table 3, no significant difference of MDA levels was observed in normal rats without or with ECE treatments (NC, N + E2 and N + E8), and alloxan induction (DC) upraised MDA levels with no significant difference only in spleen compared to that of the NC rats. In diabetic rats treated with ECE, an extremely significant difference compared to that of the NC rats was found only in liver, and significant decrease compared to that of the DC rats, but without in a dose-dependent manner, was found only in heart.

For a long time, extracts rich in polyphenolics and/or flavonoids from numerous medicinal plants have been reported to be effective against ROS-related damage by their antioxidant activities thus reduction of hyperglycemia in alloxan- or streptozotocin-induced diabetes [21,22,44,45]. Our previous study showed that ECE contained considerable phenolics, mainly ellagic acid, kaempferol and their derivatives as mentioned above [13], and exhibited high antioxidant activities [13]. Phenolics are good for scavenging various free radicals leading to reduced lipid peroxidation. The present in vivo study indicates that ellagic acid, kaempferol and their derivatives enriched in ECE might reduce MDA levels in organs such as heart and liver through acting as strong antioxidants.

#### 2.4.3. Effects of ECE on SOD Activities

There are considerable evidences that oxidative stresses would be increased in diabetes [5]. Elevated concentration of free radicals and increased lipid peroxidation would inevitably result in decrease of antioxidant defense ability in biological systems. Therefore, the presence of certain levels of vigorous antioxidants such as SOD are vital to overcome various oxidative stresses [2]. As shown in Table 4, with a significant increase of the SOD activity in heart with ECE treatment at the higher dosage (N + E8) as an only exception, no significant changes were observed in all the four organs of all the other normal groups of rats compared to that of NC rats. SOD activities of the DC rats were decreased significantly in heart and spleen and extremely significantly in liver and kidney compared to those of NC rats, and ECE administration to DC rats at both the two dosages (i.e., D + E2 and D + E8) significantly recovered SOD activities to a similar extent in all the four organs, with three organs (heart, kidney and spleen) being restored back to the levels of the NC rats and one organ (liver) even became extremely significantly higher than that of the NC rats. These results suggest that ECE could also ameliorate the alloxan-induced type 1 diabetes via improving the reduced SOD activities in DC rats, especially its SOD activity in liver. Furthermore, degradation of antioxidant enzymes such as SOD, catalase and glutathione peroxidase, and their associated co-factors are known to be essential for decrease of pancreatic insulin secretion [46]. Therefore, the recovered or even enhanced (e.g, in liver) SOD activity by ECE might contribute to increase of pancreatic β-cell viability of diabetic rats to secret insulin.

## 3. Materials and Methods

### 3.1. Reagents and Animals

Alloxan and tolbutamide were purchased from Sigma-Aldrich Chemical (St. Louis, MO, USA). Serum triglyceride, total cholesterol, high density lipoprotein (HDL), low density lipoprotein (LDL) and malondialdehyde (MDA) assay kits were bought from Abcam (Cambridge, MA, USA). Glutathione (GSH) and superoxide dismutase (SOD) assay kits were bought from BioVision (Milpitas, CA, USA). Aspartate aminotransferase (AST) assay kit was purchased from Abnova (Taipei, Taiwan). All other chemicals and reagents were of analytical grade. Male SD rats were purchased from the Laboratory Animal Center of the Academy of Military Medical Sciences (Beijing, China), and they were housed under a temperature controlled at 25 ± 1 °C on a 12-h light/dark cycle. The rats were fed on pelleted food, and tap water was available *ad libitum*. All experimental rats were in compliance with the ethical recommendations and guidelines for the care of laboratory animals and the animal permit number is SYXK（Beijing）2016-0005. 

### 3.2. Preparation of Ethanol Crude Extract from Mongolian Oak Cups

Mongolian oak (*Quercus mongolica* Fisch. ex Ledeb.) cups were collected from Grand Khingan in Heilongjiang Province and authenticated by Dr. Zhonghua Liu (Beijing Forestry University, Beijing, China). The oak cups were air-dried until equilibrium humidity, ground and screened through a 1-mm mesh, then stored in darkness at room temperature for further use.

Ethanol crude extract (ECE), namely, crude extract prepared with 50% ethanol from Mongolian oak cups, was prepared according to our previous report [13]. In brief, oak cup powder (100 g) was extracted three times by heating to reflux at 80 °C with 50% aqueous ethanol (1000 mL), each for 1 h. The combined supernatants (3000 mL) were concentrated on a rotary evaporator, then dried in a water bath set at 60 °C to prepare ECE. The prepared ECE was stored in a −20 °C refrigerator for further use. Prior to oral administration to the experimental rats, the ECE was suspended uniformly in distilled water.

### 3.3. Induction of Experimental Diabetes

Diabetic rats were induced by intraperitoneal administration of alloxan. Prior to experiments, rats (220–240 g) were fed with standard rodent food for one week for acclimation to the lab conditions. The acclimated rats were fasted for 12 h with free access to water, then injected with 0.5% alloxan dissolved in 10 mM sodium citrate (pH 4.5) at a dose of 150 mg/kg. The normal rats received an injection of 10 mM sodium citrate (pH 4.5). Diabetic rats were confirmed by the fasting blood glucose concentration above 15 mmol/L at the 7th day after alloxan administration, together with polyphagia, polydipsia, polyuria, and body weight loss.

### 3.4. Experimental Design

Diabetic and normal rats were randomly divided into seven groups (*n* = 5): (1) normal control (NC): normal rats treated with normal saline per day, 5 mL/kg BW; (2) normal + 200 mg/kg ECE (N + E2): normal rats treated with ECE at 200 mg/kg per day, 5 mL/kg BW; (3) normal + 800 mg/kg ECE (N + E8): normal rats treated with ECE at 800 mg/kg per day, 5 mL/kg BW; (4) diabetes control (DC): diabetic rats treated with normal saline per day, 5 mL/kg BW; (5) diabetes + tolbutamide (D + T): diabetic rats treated with tolbutamide at a dose of 100 mg/kg per day, 5 mL/kg BW; (6) diabetes + 200 mg/kg ECE (D + E2): diabetic rats treated with ECE at 200 mg/kg per day, 5 mL/kg BW; (7) diabetes + 800 mg/kg ECE (D + E8): diabetic rats treated with ECE at 800 mg/kg, 5 mL/kg BW. Doses of ECE were determined according to a preliminary experiment (data not shown). ECE, saline and tolbutamide were orally administrated by gavage once a day for 15 consecutive days. Fasting blood glucose level and body weight were determined on days of the 2nd, 4th, 7th, 10th and 15th after the ECE administration. On the 15th day, animals of all groups were anaesthetized by giving an intraperitoneal injection of 40 mg/kg pentobarbital sodium and blood serum of each rat was collected. Heart, liver, spleen and kidney of each animal were collected for determination of organ indexes and antioxidant indicators. Organ index = *W*_0_/*W_b_*, where *W*_0_ is the organ weight and *W_b_* is the body weight of an individual rat. Blood of each rat was gathered to measure levels of serum triglyceride, total cholesterol, HDL cholesterol, LDL cholesterol, and AST.

### 3.5. Blood Tests of Lipid Profile and AST Level

Fasting blood samples were collected from the tip of tail veins of rats after oral administration of ECE for 15 consecutive days. The blood was used for determination of glucose concentration with a glucometer (GLUCOCARD Test Strip II, Tokushima, Japan) immediately after collection. Levels of serum triglyceride, total cholesterol, HDL and LDL cholesterol, and AST in the collected blood samples were determined using corresponding assay kits. All kits were used by following the manufacturer’s instruction, and all results were expressed as mmol per liter of serum (mmol/L), with AST expressed as KarU as an exception.

### 3.6. Determination of Three Antioxidant Indicators

Determination of MDA, GSH and SOD levels in heart, liver, spleen and kidney tissues of normal and diabetic rats without and with ECE treatments were performed by following instructions of their corresponding commercial kits. Results of these three measurements were expressed as μmol/mg protein, μmol/g protein and kNU/g protein, respectively.

### 3.7. Statistical Analysis

Data were presented as mean ± SD of three parallel measurements. Statistical significances (*t*-test: two-sample equal variance, using two-tailed distribution) were determined by using Microsoft Excel 2016 (Redmond, WA, USA). *p* < 0.05 and 0.01 were set to be significant and extremely significant, respectively.

## 4. Conclusions

**Figure 4:**
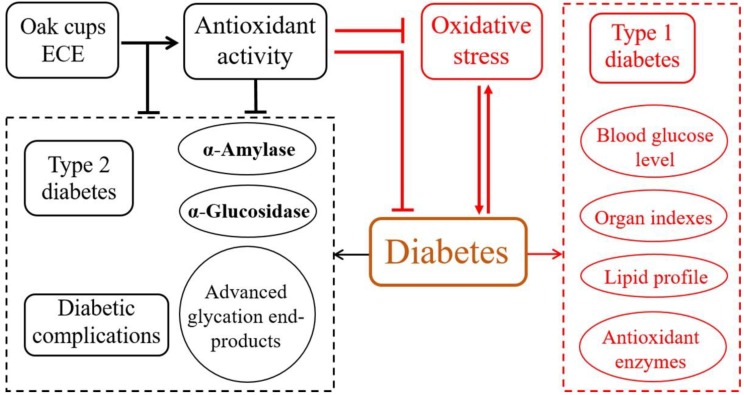
Proposed hypoglycemic mechanisms of ECE prepared from Mongolian oak cups. The proposed mechanism within the black dashed box on the left was summarized based on data of an in vitro study in one of our previous reports [1], and that within the red dashed box on the right was proposed according to the present in vivo investigation.

Mongolian oak is distributed broadly in northern China. The current study investigated the hypoglycemic effects of phenolic extracts from Mongolian oak cups (i.e., ECE) in normal and alloxan-induced diabetic rats. The results show that after oral administration of ECE for 15 consecutive days at 800 mg/kg, fasting blood glucose levels, indexes of heart and liver, and levels of cholesterol, triglyceride and MDA in heart were significantly decreased, and HDL levels and SOD activities in both heart, liver, spleen and kidney in diabetic rats were significantly improved. ECE treatment at 200 mg/kg for 15 days could also improve the situation of diabetic rats to certain degrees. These anti-diabetic activity of ECE might result from its high contents of ellagic acid, kaempferol and their derivatives, and its superior antioxidant capacity. On the other hand, in normal rats, no significant differences were exhibited in both fasting blood glucose levels, body weight, indexes of heart, liver, spleen and kidney, and levels of cholesterol, triglyceride, LDL and AST in serum and GSH and MDA in all the four tissues after ECE administration. Taking together with our previous in vitro study that ECE could also inhibit α-amylase, α-glucosidase and diabetes complications [1,12], we propose that ECE enriched in ellagic acid, kaempferol and their derivatives from Mongolian oak cups could exert anti-diabetic effects through alleviating types 1 and 2 diabetes and their complications (Figure 4). Therefore, oak cups, an abundant and low-cost by-products, possessed great potential to be explored as a source of natural anti-diabetic agents. The results would extend our knowledges on potential bioactivities and pharmaceutical applications of ECE. Nevertheless, ECE administration also decreased HDL and increased LDL levels in normal rats, suggesting that further research is needed to assess its security.

## Figures and Tables

**Table 1:** Effects of ECE on lipids and lipoprotein of normal and diabetic rats (mmol/L).

Group	Cholesterol	Triglyceride	HDL	LDL
NC	1.562 ± 0.319	0.286 ± 0.057	1.730 ± 0.466	0.407 ± 0.033
N + E2	1.517 ± 0.258	0.548 ± 0.058	0.742 ± 0.142 ^#^	0.735 ± 0.045 ^##^
N + E8	1.385 ± 0.116	0.354 ± 0.110	0.745 ± 0.104 ^#^	0.677 ± 0.230 ^##^
DC	2.760 ± 0.198 ^##^	2.001 ± 0.380 ^##^	1.107 ± 0.018 ^#^	0.793 ± 0.186 ^#^
D + T	3.056 ± 0.177 ^##^	2.057 ± 0.163 ^##^	1.553 ± 0.094 *^#^	0.742 ± 0.179 ^#^
D + E2	2.459 ± 0.383 ^#^	1.134 ± 0.244 ^##^*	1.243 ± 0.157 ^#^	0.728 ± 0.164 ^#^
D + E8	1.947 ± 0.453 *	0.860 ± 0.563 *	1.670 ± 0.303 *	0.648 ± 0.158 ^#^

Results were presented as mean ± SD with five independent experiments (*n* = 5). ^#^ and ^##^ indicate significant (*p* < 0.05) and extremely significant (*p* < 0.01) differences between the normal controls (NC) and all other groups, and * indicates significant (*p* < 0.05) differences between the diabetic controls (DC) and all other diabetic rats (D + T, D + E2 and D + E8), respectively. N, normal; C, control; D, diabetes; T, tolbutamide at 100 mg/kg; E2, ECE at 200 mg/kg; E8, ECE at 800 mg/kg.

**Table 2:** Effects of ECE on GSH levels in organs of normal and diabetic rats.

Group	GSH ± SEM (μmol/g prot)			
	Heart	Liver	Kidney	Spleen
NC	0.50 ± 0.10	2.53 ± 0.30	1.10 ± 0.12	5.68 ± 0.14
N + E2	0.47 ± 0.09	2.50 ± 1.35	1.02 ± 0.44	5.70 ± 1.06
N + E8	0.48 ± 0.19	2.72 ± 0.37	1.08 ± 0.13	5.78 ± 0.11
DC	0.59 ± 0.04 ^#^	2.74 ± 0.23	1.38 ± 0.33	9.98 ± 4.54 ^#^
D + T	0.48 ± 0.20	1.82 ± 0.29 *^#^	1.51 ± 0.32	7.12 ± 0.40 ^#^
D + E2	0.37 ± 0.12 **	2.52 ± 0.36	1.43 ± 0. 36	6.62 ± 1.56
D + E8	0.32 ± 0.04 **^#^	1.78 ± 0.29 *^#^	1.17 ± 0.27	7.33 ± 1.69

Results were presented as mean ± SD with five independent experiments (*n* = 5). * and ** indicate significant (*p* < 0.05) and extremely significant (*p* < 0.01) differences between the diabetic controls (DC) and all other diabetic rats (D + T, D + E2 and D + E8), and ^#^ indicates significant (*p* < 0.05) differences between the normal controls (NC) and all other groups of rats, respectively. N, normal; C, control; D, diabetes; T, tolbutamide at 100 mg/kg; E2, ECE at 200 mg/kg; E8, ECE at 800 mg/kg.

**Table 3:** Effects of ECE on MDA levels in organs of normal and diabetic rats.

Group	MDA ± SEM (μmol /mg prot)			
	Heart	Liver	Kidney	Spleen
NC	0.94 ± 0.17	1.20 ± 0.40	0.12 ± 0.06	1.24 ± 0.80
N + E2	0.93 ± 0.40	1.41 ± 0.95	0.10 ± 0.03	1.26 ± 0.11
N + E8	0.78 ± 0.08	1.19 ± 0.12	0.13 ± 0.01	1.07 ± 0.22
DC	1.22 ± 0.48 ^#^	3.54 ± 1.32 ^##^	0.17 ± 0.04 ^#^	1.31 ± 0.13
D + T	0.60 ± 0.51 *	3.10 ± 0.48 ^##^	0.08 ± 0.04 *	1.04 ± 0.65
D + E2	0.91 ± 0.06 *	3.40 ± 1.64 ^##^	0.12 ± 0.03	1.27 ± 0.19
D + E8	0.94 ± 0.05 *	2.95 ± 0.43 ^##^	0.14 ± 0.02	1.25 ± 0.26

Results were presented as mean ± SD with five independent experiments (*n* = 5). * indicates significant (*p* < 0.05) differences between the diabetic controls (DC) and all other diabetic rats (D + T, D + E2 and D + E8), and ^#^ and ^##^ indicate significant (*p* < 0.05) and extremely significant (*p* < 0.01) differences between the normal controls (NC) and all other groups of rats, respectively. N, normal; C, control; D, diabetes; T, tolbutamide at 100 mg/kg; E2, ECE at 200 mg/kg; E8, ECE at 800 mg/kg.

**Table 4:** Effects of ECE on SOD activities in organs of normal and diabetic rats.

Group	SOD ± SEM (kNU/g prot)			
	Heart	Liver	Kidney	Spleen
NC	117.46 ± 7.37	115.61 ± 17.05	118.66 ± 5.63	130.00 ± 17.96
N + E2	126.77 ± 14.16	119.21 ± 30.49	115.98 ± 9.76	121.22 ± 26.09
N + E8	141.75 ± 6.56 ^#^	114.57 ± 28.75	116.83 ± 15.93	131.48 ± 6.22
DC	99.27 ± 3.28 ^#^	106.76 ± 6.27 ^##^	89.46 ± 12.02 ^##^	96.02 ± 4.49 ^#^
D + T	105.48 ± 11.15	102.69 ± 13.15	103.94 ± 34.42 *	118.43 ± 16.59 *
D + E2	111.08 ± 19.69 *	121.07 ± 8.95 *^##^	102.98 ± 22.98 *	122.87 ± 31.40 *
D + E8	108.19 ± 14.81 *	118.82 ± 21.77 *^##^	113.18 ± 19.02 *	125.14 ± 13.39 *

Results were presented as mean ± SD with five independent experiments (*n* = 5). * indicates significant (*p* < 0.05) differences between the diabetic controls (DC) and all other diabetic rats (D + T, D + E2 and D + E8), and ^#^ and ^##^ indicate significant (*p* < 0.05) and extremely significant (*p* < 0.01) differences between the normal controls (NC) and all other groups of rats, respectively. N, normal; C, control; D, diabetes; T, tolbutamide at 100 mg/kg; E2, ECE at 200 mg/kg; E8, ECE at 800 mg/kg.
